# Immunology and Genetic of Preeclampsia

**DOI:** 10.1080/17402520600876903

**Published:** 2006

**Authors:** Norma C. Serrano

**Affiliations:** Biomedical Research Centre Universidad Autónoma de Bucaramanga (UNAB) Bucaramanga Colombia

## Abstract

Preeclampsia is a disease characterized by hypertension and proteinuria in the third trimester of pregnancy. Preeclampsia is a major cause of maternal mortality, and fetal death, especially in developing countries, but its aetiology remains unclear. Key findings support a causal role of superficial placentation driven by immune mal maladaptation, which then lead to reduced concentrations of angiogenic growth factors and to an increase in placental debris in the maternal circulation resulting in a maternal inflammatory response. Epidemiological research has consistently demonstrated a substantial familial predisposition to preeclampsia. Unfortunately, the conquest of the genes explaining such a individual susceptibility has been proved to be a hard task. However, genetics will also inform us about causality of environmental factors, and then serve as a tool to prioritize therapeutic targets for preventive strategies.


*Clinical & Developmental Immunology*, June–December 2006; 13(2–4): 197–201

Taylor & Francis

Taylor & Francis Group

### Immunology and genetic of preeclampsia

NORMA C. SERRANO

*Biomedical Research Centre, Universidad Autónoma de Bucaramanga (UNAB), Bucaramanga, Colombia*

**Abstract**
Preeclampsia is a disease characterized by hypertension and proteinuria in the third trimester of pregnancy. Preeclampsia is a major cause of maternal mortality, and fetal death, especially in developing countries, but its aetiology remains unclear. Key findings support a causal role of superficial placentation driven by immune mal maladaptation, which then lead to reduced concentrations of angiogenic growth factors and to an increase in placental debris in the maternal circulation resulting in a maternal inflammatory response. Epidemiological research has consistently demonstrated a substantial familial predisposition to preeclampsia. Unfortunately, the conquest of the genes explaining such a individual susceptibility has been proved to be a hard task. However, genetics will also inform us about causality of environmental factors, and then serve as a tool to prioritize therapeutic targets for preventive strategies.

**Keywords:** *Preeclampsia, trofoblast, HLA, cytokines, candidate gene*

### Introduction

Preeclampsia is a multisystemic disorder of unknown aetiology, which occurs in around 5% of all pregnancies. Preeclampsia can be manifested as a maternal syndrome (hypertension, proteinuria with or without other multi-systemic alterations), or as a foetal syndrome (foetal growth restriction, diminished amniotic fluid, and altered oxygenation) (Sibai et al. 2005).

Preeclampsia has a big deleterious impact in the maternal and foetus morbidity and mortality worldwide, but mainly in developing countries. A recent, systematic review indicated that hypertensive pregnancy disorders are the leading cause of maternal death in Latin American and Caribbean countries (25.7%, 7.9–52.4) (Khan et al. 2006).

The aetiology of preeclampsia is still unknown, but it is accepted that susceptibility for its development is given by the presence of complex gene–gene and gene–environment interactions between the mother and the foetus. Previous studies observational studies have showed compelling evidence of the presence of risk factors for preeclampsia such as nulliparity, family history of preeclampsia or eclampsia, personal history of a previous pregnancy with preeclampsia, increment in the trophoblastic mass (multiple pregnancy, molar pregnancy), paternity change between pregnancies, age over 40 years, obesity, and some maternal chronic conditions such as diabetes, chronic hypertension, renal disease, autoimmune disease, antiphospholipid syndrome, while other factors as smoking during pregnancy, regular physical activity and prenatal C and E vitamin ingestion have been considered as potential protective factors (Duckitt and Harrington 2005; Sibai et al. 2005). However, recent clinical trials using vitamin C and E supplementation, have called into question some of these associations (Poston et al. 2006).

Nevertheless, considerable progress in the last decade has led to a better understanding of the physiopathology of preeclampsia, which is supposed to be determined by two essential processes; the first given by a superficial trophoblastic invasion and a poor remodeling of the spiral arteries and of the maternal decidua, leading to an inadequate placentation and therefore to a poor placental oxygenation, and an hypoxic environment. Findings that are, perhaps, the responsible of the vasoconstriction and an increased maternal arterial blood pressure, but also may be

Correspondence: N.C. Serrano, Biomedical Research Centre, Universidad Autónoma de Bucaramanga (UNAB), Bucaramanga, Colombia. Tel: 57 76399151. Ext. 139. Fax: 57 76399147. E-mail: nserrano@unab.edu.co

ISSN 1740-2522 print/ISSN 1740-2530 online © 2006 Taylor & Francis
DOI: 10.1080/17402520600876903

responsible for triggering a complex cascade of events that would lead to the endothelial dysfunction observed during the preeclampsia and all its deleterious consequences (Roberts and Hubel 1999; Saftlas et al. 2005). A substantial part of this process could be consequence of an abnormal immune response to the antigenic stimulus given by the foetal–placental graft (Saftlas et al. 2005) (Figure 1).

### Cytokines and preeclampsia

A normal pregnancy is accompanied by an inflammatory response, which is exaggerated in the case of preeclampsia, and it has been postulated that endothelial dysfunction can be a part of this exacerbated phenomenon (Roberts and Hubel 1999). Th1-type cytokines such as α-tumoral necrosis factor (TNF-α), γ-interferon (IFN-γ) and interleukin 2 (IL-2) induce trophoblastic apoptosis, restrain differentiation and invasion of trophoblast, and thus may be involved in the incomplete invasion of trophoblast to spiral arteries and shadow implantation of placenta that are integral pathologies of preeclampsia (Dong et al. 2005). On the other side, Th2 cytokines such as IL-4 and IL-10, stimulate the differentiation and proliferation of the trophoblast; low levels of IL-10 have been reported on women with preeclampsia compared to pregnant women with normal arterial blood pressure, so high levels of Th1 and low levels of Th2 cytokines, through a Th41/Th2 imbalance, could lead to a functional injury of the trophoblast in preeclampsia (Dong et al. 2005; Makris et al. 2006).

The exaggerated inflammatory response towards pregnancy could be a condition of genetically susceptible women (Redman et al. 1999). A study performed in white and African–American women gathered in the USA, found that white women with preeclampsia had a higher probability of carrying the genotype FNT-α −308A/A than white pregnant women with normal blood pressure (odds ratio [OR]: 4.1; 95%CI: 1.1–15.3), but no association was found in African–American women. Both white and African–American women had with preeclampsia had a higher probability of carrying the genotypes for IL-1α −4845G/G (African–American women's OR: 11.7; white women's OR 1.9) and −899C/C (African–American women's OR: 5.9; white women's OR 2.3). No significant differences regarding the allelic distribution were found between preeclamptic women and pregnant women with normal blood pressure for the −1082A IL-10 polymorphism (Haggerty et al. 2005). Another recent study performed in white and African–American women from Brazil, analysed the possible association between preeclampsia and the −308

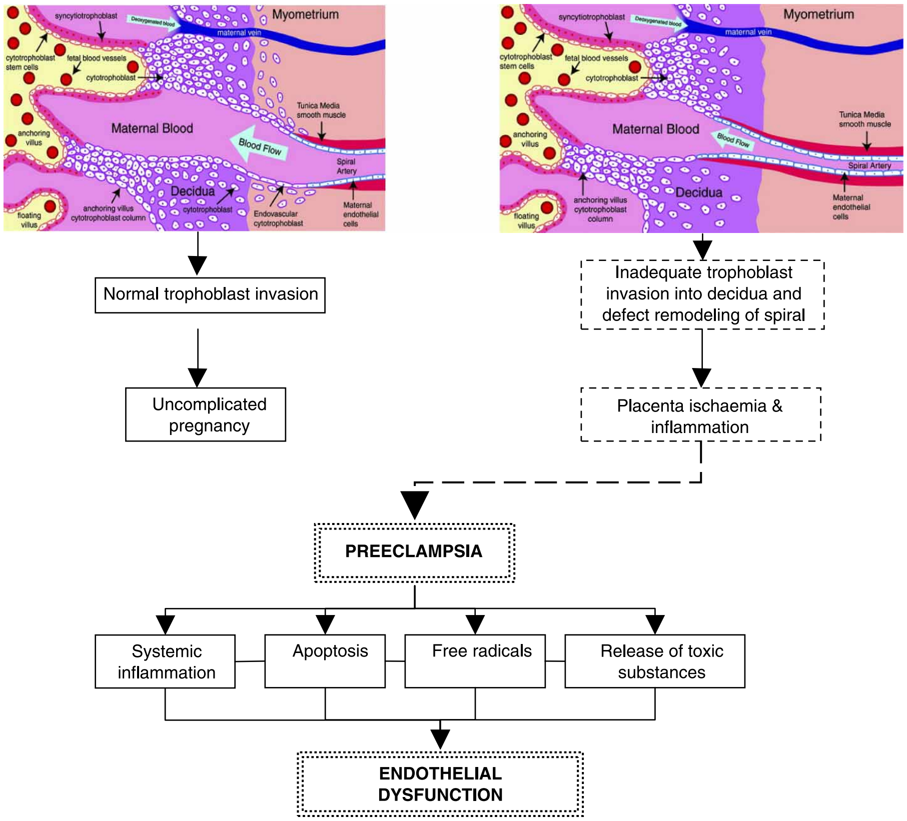

Figure 1. Mechanisms of placental development in uncomplicated pregnancies and of pathological placentation, as in preeclampsia.

TNFα, transforming growth factor-$\beta_1$ (+10; 25), −1082 IL-10, −174 IL-6, and +874 interferon-γ polymorphisms, observing a lower frequency for the IL-10 −1082G/G genotype in women with preeclampsia than in the control group, and a lack of association with the other studied polymorphisms including the results of the meta-analysis for the TNF-α (Daher et al. 2006)

### HLA and preeclampsia

During pregnancy, the maternal immune system gets in direct contact with the cells and tissues of the semiallogenic foetus graft and so, mechanisms must exist to modulate and moderate the immune maternal response to such stimulus. The trophoblastic cells, originated in the foetus, do not express classic human leukocyte antigens (HLA) of the Ia and II classes, except for a weak expression of HLA-C, and they express non-classic antigens of the Ib, HLA-G, -F and -E classes (Saftlas et al. 2005; Ishitani et al. 2003), so these molecules could be involved in certain pregnancy complications such as preeclampsia, intrauterine growth restriction, and recurrent abortion. The HLA-G can inhibit T and NK-mediated cellular lysis, through direct interaction with the ILT2, ILT4 and KIR2DL4 receptors, so cells from the invasive trophoblast that do not express HLA-Ib on their surface would be exposed to NK-mediated cellular lysis; the strong expression of HLA-G molecules by the invasive trophoblast joined to the expression of HLA-E and HLA-F in the placenta, could prevent this event. Proteins coded by HLA-G are almost monomorphic, finding that markedly contrasts with the Ia and II classes of HLA, which are highly polymorphic. Regarding the HLA-G, polymorphisms have been reported in both the regulator region on the 5′ end (5′URR) and the non-transcribed 3′ region of the gene (3′UTR). Fifteen alleles have been recognized by the WHO nomenclature committee for factors in the HLA system; nevertheless, only five HLA-G proteins with simple aminoacid substitutions have been described in the literature, two of them a product of nucleotide substitutions in exon 2 (defining the G*0101 and G*0103 alleles), one in exon 3 (defining the alleles G*1040 × ), and another one in exon 4 (defining the g*0106 allele) (Hviid 2006).

Two studies have independently reported a risk significantly augmented for the development of preeclampsia in women pregnant for the first time, when the foetuses are carriers of the +14/+14 bp HLA-G, postulating that this genotype could lead to a diminished expression of HLA-G in the placenta (Hviid 2006). Even so, the results have not been reproducible in other studies; therefore more evidence is required to clear the possible role of HLA-G in the pathogenesis of preeclampsia.

### Inheritance, genes and preeclampsia

Preeclampsia has a great genetic component determined by multiple genes, which has been evidenced through twin concordance studies and family aggregation studies. A study developed in Swedish population including monozygotic (MC) and dicygotic (DC) twins classified by 13 polymorphic markers, found a concordance for preeclampsia of 0.57 in MC twins and 0.18 in DC twins, and a heritability of 54% (Salonen et al. 2000)

Recently Nilsson et al. (2004), in a large family aggregation study for preeclampsia with 1,188,207 live births in Swedish population analysed under a quantitative genetics model, found that the genetic effect was responsible for 31% (95%CI 9–45) of the disease burden, while the non-shared environmental effect was only responsible for 63% (95%CI 55–74) and the common environmental effect was very low and not significant.

For complex diseases, the genome-wide scan strategy has been used, with the aim of identifying susceptibility loci for the development of the disease. Genome scans developed in families with preeclampsia from Australia, New Zealand, Iceland and Finland have localized in chromosome 2 possible susceptibility loci for the development of preeclampsia/eclampsia. The mapped locus in the population from Australia and New Zealand was named PREG1 and is possibly the same locus found in Finnish population. A posterior study using the fine mapping strategy and SNP analysis determined, in the PREG1 locus, two significant linkage peaks, 2p11 and 2p23. Analysing the public databases, two genes located in those regions and related to the physiopathology of preeclampsia were chosen for the SNP analysis (Fitzpatrick et al. 2004); the TACR1 gene which codes for a vasoactive neuropeptide, neurokinin B (NKB), and which concentration has been found elevated in pregnancies complicated with preeclampsia compared to normal pregnancies (Page et al. 2000), and the TCF7L1 gene, which codes for a transcription factor that functions as a mediator in the WNT signalling pathway and is antagonised by the β-transforming growth factor signalling pathway. The WNY signalling pathway contributes to the establishment of the relationship between the blastocist and the luminal uterine epithelium, and to the synthesis of the extra-cellular matrix components associated to decidualization (Paria et al. 2001).

Six SNPs for the TACR1 gene and five for the TCF7L1 gene were genotyped in a group of families that showed linkage in the fine mapping analysis, two SNPs showed significant LOD scores, higher than 3, one for each studied gene. Although, no evidence of linkage disequilibrium (LD) was found under any of the heredity models for any of the SNPs (Fitzpatrick et al. 2004).

Table I. Candidate genes studies in preeclampsia (Chappell and Morgan 2006).

| Gene name | Gene symbol | Gene name | Gene symbol |
|---|---|---|---|
| Trombophilia | | Cytokines | |
| Factor V Leiden | F5 | α-tumoral necrosis factor | TNF |
| Prothrombin 20210 | F2 | IGF II | IGF2 |
| Methylene tetrahydrofolate reductase | MTHFR | Interleukin Iα | ILIA |
| Cystathione β-synthase | CBS | Interleukin Iβ | ILIB |
| Plasminogen activatior inhibitor I | SERPINEI | Interleukin 10 | IL10 |
| β-Fibrinogen | FGB | T-lymphocyte-associated protein 4 | CTLA4 |
| Platelet glycoprotein IIIa | ITGB3 | TNF-receptor superfamily member 6 | FAS |
| Trombomodulin | THBD | Oxidative stress | |
| Factor VII | F7 | Microsomal epoxide hydrolase | EPHXI |
| Platelet collagen receptor α2β1 | ITGA2 | Glutathione *S*-transferase pi | GSTPI |
| Factor XIII A-subunit | FI3AI | Glutathione *S*-transferase mu I | GSTMI |
| Haemodynamics | | Glutathione *S*-transferase theta I | GSTTI |
| Angiotensionogen | AGT | Myeloperoxidase | MPO |
| Renin | REN | Maganese supeoxide dismutase | SOD2 |
| Angiotensin-coverting enzyme | ACE | Cytochrome IAI | CYPIAI |
| ATI receptor | AGTR1 | Hepatoglobin | HP |
| AT2 receptor | AGTR2 | $P22^{phox}$ | CYBA |
| Epithelial sodium channel | SCNNIB | Lipid metabolism | |
| Endothelial function | | Lipoprotein lipase | LPL |
| Nitic oxide synthase endothelial | NOS3 | Apolipoprotein E | APOE |
| Endothelin I | EDNI | Peroxisome-proliferator-activated receptor γ | PPARG |
| Dimetthylarginine dimethylaminohydrolase 2 | DDH2 | Cholesteryl ester transfer protein | CETP |
| G-protein β3 | GNB3 | $\beta_3$-Adrenergic receptor | ADRB3 |
| Endocrine | | Angiogenesis | |
| Oestrogen receptor α | ESRI | Vascular endothelial growth factor | VEGF |
| Oestrogen receptor β | ESR2 | Matrix mitallopeptidase I | MMPI |
| Inhibin α | INHA | | |

The number of genes involved in the susceptibility to develop preeclampsia increases rapidly, specially those genes found involved with the maternal homeostatic and cardiovascular system, or related to the regulation of the maternal inflammatory response.

Nearly 50 maternal genes have been analysed; most of these studies have been approaches to identify a genetic association, comparing the frequencies of genetic polymorphisms between cases and controls. Although close to 50 different genes have been studied, 70% of the publications revolve around eight candidate genes, which have been analysed because of their biologic plausibility in preeclampsia. Among them there are genes that code for the rennin–angiotensin system which regulate blood pressure (angiotensinogen, angiotensin converting enzyme, and angiotensin receptor), inherited thrombophilias (Factor V Leiden V, prothrombin, and metilene-tetrahydrofolate reductase), vasodilatation regulating genes (endothelial nitric oxide synthase, eNOS) and genes that code for pro-inflammatory cytokines (FNT-α) (Chappell and Morgan 2006) (Table I). In a large study from Colombia, we found that the eNOS Glu298Asp polymorphism is associated with a significant increased risk of preeclampsia ($p = 0.001$) (Serrano et al. 2004).

### Conclusion

Preeclampsia is a multisystemic disorder based in a cascade of immunogenetic events apparently originated from an inadequate placentation. A unique mechanism that explains the complexity of the pathogenesis exists, and given so, neither does a single marker that can predict the beginning of the disease. Nevertheless, research strongly points to the recognition of immunologic, biochemical and genetic markers that could contribute to a better understanding of the disease and in final to its prevention.

### References

Chappell S, Morgan L. 2006. Searching for genetic clues to the causes of pre-eclampsia. Clin Sci 110:443–458.

Daher S, Sass N, Olivera LG, Mattar R. 2006. Cytokine genotyping in preeclampsia. Am J Reprod Immunol 55:130–135.

Dong M, Zhengping JH, Xie X, Wang H. 2005. Placental imbalance of Th-1 and Th-2-type cytokines in preeclampsia. Acta Obstet Gynecol Scand 84:788–793.

Duckitt K, Harrington D. 2005. Risk factors for pre-eclampsia at antenatal booking: Systematic review of controlled studies. BMJ 12; 330(7491):565.

Fitzpatrick E, Göring HHH, Liu H, Borg A, Forrest S, Cooper DW, Brennecke SP, Moses EK. 2004. Fine mapping and SNP analysis of positional candidates at the preeclampsia susceptibility locus (PREG1) on chromosome 2. Hum Biol 76:849–862.

Haggerty CL, Ferrell RE, Hubel CA, Markovic N, Harger G, Ness R. 2005. Association between allelic variants in cytokine genes and preeclampsia. Am J Obstet Gynecol 193:209–215.

Hviid TVF. 2006. HLA-G in human reproduction: Aspect of genetics, function and pregnancy complications. Hum Reprod Update 12:209–232.

Ishitani A, Sageshima N, Lee N, Dorofeeva N, Hatake K, Marquardt H, Geraghty DE. 2003. Protein expression and peptide binding suggest unique and interacting functional roles for HLA-E, F and G in maternal placental immune recognition. J Immunol 171:1376–1384.

Khan K, Wojdyla D, Say L, Gülmezoglu MA, A Van Look PF. 2006. WHO analysis of causes of maternal death: A systematic review. Lancet 367:1066–1074.

Makris A, Yu B, Thorthon C, Hennessy A. 2006. Placental deficiency of interleukin-10 (IL-10) in preeclampsia and its relationship to an IL10 promotor polymorphism. Placenta 27: 445–451.

Nilsson E, Salonen Ros H, Cnattingius S, Lichtenstein P. 2004. The importance of genetic and environmental effects for pre-eclampsia and gestational hypertension: A family study. BJOG 111:200–206.

Page NM, Woods RJ, Gardiner SM, Lomthaisong K, Gladwell RT, Butlin DJ, Manyonda IT, Lowry PJ. 2000. Excessive placental secretion of neurokinin B during the third trimester causes pre-eclampsia. Nature 405(6788):797–800, 15 June.

Paria BC, Ma W, Tan J, Raja S, Das SK, Dey SK, Hogan BL. 2001. Cellular and molecular responses of the uterus to embryo implantation can be elicited by locally applied growth factors. Proc Natl Acad Sci USA 30; 98:1047–1052.

Poston L, Briler AL, Seed PT, Kelly FJ, Shennon AH. 2006. Vitamin C and vitamin E in pregnancy women at risk for pre-eclampsia (VIP trial): Randomized placebo-controlled trial. Lancet 3367:1145–1154.

Redman CW, Sacks GP, Sargent IL. 1999. Preeclampsia: An excessive maternal inflammatory response to pregnancy. Am J Obstet Gynecol 180:499–506.

Roberts JM, Hubel CA. 1999. Is oxidative stress the link in the two-stage model of preeclampsia? Lancet 354:788–789.

Saftlas AF, Beydoun H, Triche E. 2005. Immunogenetic determinants of preeclampsia and related pregnancy disorders. A systematic review. Obstet Gynecol 106:162–172.

Salonen RH, Lichtenstein P, Lipworth L, Cnattingius S. 2000. Genetic effects on the liability of developing pre-eclampsia and gestational hypertension. Am J Med Genet 91:256–260.

Serrano N, Casas JP, Díaz LA, Páez MC, Mesa CM, Cifuentes Monterrosa A, Bautista A, Hawe E, Hingorani A, Vallance P, López-Jaramillo P. 2004. Endothelial nitric oxide synthase genotype and risk of preeclampsia: A multi-centre case-control study. Hypertension 44:702–707.

Sibai BM, Dekker G, Kupferminc M. 2005. Pre-clampsia. Lancet 365:785–799.

